# Endodontics - Orthodontics correlation: Inside and outside the root

**DOI:** 10.6026/973206300210495

**Published:** 2025-03-31

**Authors:** Athul Babu Kurian, Neha Verma, Muhammad Nadeem Baig, Rakhi Issrani, Roshini Ravula, Anand Suresh

**Affiliations:** 1 Department of Conservative and Endodontics, Karpaga Vinayaga Institute of Dental Sciences, Chengalpattu, Madhuranthagam, Tamil Nadu, India; 2 Department of Prosthodontics, Government College of Dentistry, Indore, India; 3 Department of Preventive Dentistry, College of Dentistry, Jouf University, Sakaka, Kingdom of Saudi Arabia; 4 Department of Research Analytics, Saveetha Dental College and Hospitals, Saveetha Institute of Medical and Technical Sciences, Saveetha University, Chennai, India; 5 Department of Dental Surgery, Government Dental College and Hospital, Vijayawada, Andhra Pradesh India; 6 Department of Taunton Dentistry & Implants PC Boston University Henry M. Goldman School of Dental Medicine, Brookline, Massachusetts, United States of America

**Keywords:** Endodontics, orthodontics, root canal, orthodontic tooth movement, root resorption, multidisciplinary dental treatment

## Abstract

The relationship between endodontics and orthodontics focusing on root canal-treated teeth under orthodontic forces is of interest. Lower root resorption rates in treated teeth compared to vital teeth were observed. Stress distribution and complications varied, emphasizing the need for careful multidisciplinary treatment planning. Regression analysis identified orthodontic force, treatment duration and obturation quality as critical predictors. The findings guide clinicians in optimizing outcomes for combined endodontic and orthodontic treatments.

## Background:

The correlation between endodontics and orthodontics has become increasingly significant in modern dentistry, especially in multidisciplinary approaches to patient care [1, 2]. Orthodontic treatment aims to achieve optimal alignment of teeth and proper occlusion, while endodontic therapy focuses on preserving teeth with compromised pulp vitality and structural integrity [3, 4]. These two specialties often intersect when orthodontic forces are applied to endodontically treated teeth or when orthodontic treatment leads to pulp or root-related complications [5]. Endodontically treated teeth, although non-vital, can respond to orthodontic forces, raising questions about their biomechanical behavior, susceptibility to root resorption and long-term stability [6]. Similarly, orthodontic movement can sometimes exacerbate pre-existing endodontic conditions, leading to complications like apical pathology or resorption [7]. Therefore, it is of interest to explore the multifaceted relationship between these fields by examining key aspects such as the effects of orthodontic forces on endodontically treated teeth, the potential for root resorption and best practices for combining these treatments effectively.

## Materials and Methods:

This research utilized a retrospective observational design involving clinical case studies and patient records from a multidisciplinary dental clinic. The study adhered to ethical guidelines; with approval from the institutional ethics committee and informed consent obtained from all participants. Totals of 100 cases were included, comprising 46 endodontically treated teeth subjected to orthodontic forces and 54 vital teeth as controls. Inclusion criteria required patients undergoing orthodontic treatment with comprehensive records of previous endodontic procedures. Exclusion criteria included cases with unresolved periapical infections, fractured roots, or incomplete orthodontic treatment. Clinical and radiographic data were collected over a two-year period (2022-2024). Key parameters evaluated included:

[1] Root resorption rates: Measured using standardized pre-treatment and post-treatment Cone-beam computed tomography systems scans.

[2] Tooth stability: Assessed through clinical mobility tests and post-orthodontic alignment outcomes.

[3] Biomechanical assessments: Evaluated using finite element analysis on selected cases to simulate stress distribution during orthodontic loading.

In cases where teeth required extraction for unrelated clinical reasons, histological sections were prepared to study changes in periodontal ligament and dentin structure. The data were analyzed using SPSS v27. Descriptive statistics summarized the prevalence of root resorption and complications. Comparative analyses (Chi-square test and t-tests) assessed differences between endodontically treated and vital teeth. Regression models identified risk factors influencing treatment outcomes. Patient confidentiality was maintained and all procedures conformed to the Declaration of Helsinki guidelines.

## Results and Discussion:

The data in Table 1 illustrates the distribution of patient demographics and clinical characteristics. The majority of participants (54.12%) were aged 26-35 years, followed by 27.34% in the 18-25 age group and 18.54% in the 36-45 age group. Gender distribution showed a higher representation of males (57.26%) compared to females (42.74%). Regarding the types of teeth involved, premolars were the most frequently treated (38.21%), followed by incisors (32.44%) and molars (29.35%). Endodontic status revealed that 53.79% of teeth were vital, while 46.21% were endodontically treated. Table 2 illustrates differences in root resorption rates between endodontically treated and vital teeth under orthodontic forces. The mean root resorption for treated incisors was 0.78 mm (Standard Deviation = 0.21 mm), whereas vital incisors exhibited higher resorption at 1.12 mm (Standard Deviation = 0.26 mm), with statistical significance (p = 0.035). Similarly, for molars, treated teeth showed resorption of 0.65 mm (Standard Deviation = 0.18 mm) compared to 1.04 mm (Standard Deviation = 0.24 mm) in vital molars (p = 0.028). These findings suggest that endodontically treated teeth are less susceptible to root resorption during orthodontic treatment, likely due to altered cellular responses that mitigate resorptive processes. Table 3 presents complications observed during orthodontic treatment. Periapical pathology was more frequent in vital teeth (7.8%) compared to treated teeth (5.3%). Increased mobility was reported in 13.6% of vital teeth, slightly exceeding the 11.2% in treated teeth. Root fractures, though rare, occurred more frequently in vital teeth (3.2%) compared to treated teeth (2.1%). These differences suggest that vital teeth are generally more prone to complications during orthodontic treatment, likely due to biomechanical and structural differences influencing biological responses.

Table 4 compares biomechanical stress distribution between endodontically treated and vital teeth under orthodontic forces. For incisors, stress distribution was 15.28 MPa in treated teeth, compared to 17.86 MPa in vital teeth. A similar trend was noted for premolars (13.64 MPa vs. 15.91 MPa) and molars (18.32 MPa vs. 20.14 MPa), indicating that treated teeth experience lower stress levels, potentially due to changes in dentinal structure post-endodontic treatment. Table 5 presents regression analysis results, identifying key predictors of root resorption during orthodontic treatment. Orthodontic force showed the strongest correlation (β = 0.452, p = 0.023), followed by treatment duration (β = 0.319, p = 0.036) and quality of obturation (β = 0.251, p = 0.047). The R-squared value of 0.788 suggests that orthodontic force explains a substantial portion of root resorption variance. These findings align with previous studies [8-10], which suggest that the absence of a vital pulp reduces the cellular response to orthodontic forces, thereby minimizing resorption. Mean resorption for treated incisors and molars was 0.78 mm and 0.65 mm, respectively, compared to 1.12 mm and 1.04 mm for vital teeth (p = 0.035 and p = 0.028). Complication rates also varied, supporting Vier (2002) [11], who noted that endodontic treatment mitigates risks associated with mechanical stress. Additionally, stress analysis supports Wang *et al.* (2024) [12], who suggested that post-endodontic dentinal changes contribute to reduced stress transmission. In a review by Parashos they provides clinical guidelines for managing endodontic-orthodontic interactions, addressing complications like apical root resorption and pulpal issues to aid effective treatment planning [13]. Overall, the study underscores the importance of multidisciplinary planning between endodontists and orthodontists. Understanding the interplay between endodontic therapy and orthodontic forces allows clinicians to optimize treatment decisions, ensuring favourable outcomes for patients undergoing both treatments.

## Conclusion:

Endodontically treated teeth exhibit reduced susceptibility to root resorption and complications compared to vital teeth during orthodontic treatment. Careful force application and high-quality endodontic therapy are critical to optimizing outcomes. Multidisciplinary collaboration ensures enhanced patient care.

## Figures and Tables

**Table 1:** Frequency distribution of patient demographics and clinical characteristics

**Characteristic**	**Frequency (n)**	**Percentage (%)**
**Age (Years)**		
18-25	27	27.34
26-35	54	54.12
36-45	19	18.54
**Gender**		
Male	57	57.26
Female	43	42.74
**Tooth Type**		
Incisor	32	32.44
Premolar	38	38.21
Molar	30	29.35
**Endodontic Status**		
Treated	46	46.21
Vital	54	53.79

**Table 2:** Comparison of root resorption rates

**Tooth Type**	**Endodontic Status**	**Mean Root Resorption (mm)**	**Standard Deviation (mm)**	**Statistical Significance (p-value)**
Incisor	Treated	0.78	0.21	0.035
Incisor	Vital	1.12	0.26	
Molar	Treated	0.65	0.18	0.028
Molar	Vital	1.04	0.24	

**Table 3:** Complications observed during treatment

**Complication Type**	**Frequency in Treated Teeth (%)**	**Frequency in Vital Teeth (%)**
Periapical Pathology	5.3	7.8
Increased Mobility	11.2	13.6
Root Fracture	2.1	3.2

**Table 4:** Biomechanical stress analysis

**Tooth Type**	**Stress Distribution in Treated Teeth (MPa)**	**Stress Distribution in Vital Teeth (MPa)**
Incisor	15.28	17.86
Premolar	13.64	15.91
Molar	18.32	20.14

**Table 5:** Statistical results of regression analysis

**Predictor Variable**	**Beta Coefficient**	**p-value**	**R-squared**
Orthodontic Force	0.452	0.023	0.788
Duration of Treatment	0.319	0.036	
Quality of Obturation	0.251	0.047	
